# Galactosylceramide Affects Tumorigenic and Metastatic Properties of Breast Cancer Cells as an Anti-Apoptotic Molecule

**DOI:** 10.1371/journal.pone.0084191

**Published:** 2013-12-31

**Authors:** Tomasz B. Owczarek, Jarosław Suchanski, Bartosz Pula, Alicja M. Kmiecik, Marek Chadalski, Aleksandra Jethon, Piotr Dziegiel, Maciej Ugorski

**Affiliations:** 1 Laboratory of Glycobiology and Cell Interactions, Ludwik Hirszfeld Institute of Immunology and Experimental Therapy, Polish Academy of Sciences, Wroclaw, Poland; 2 Department of Biochemistry, Pharmacology and Toxicology, Faculty of Veterinary Medicine, University of Environmental and Life Sciences, Wroclaw, Poland; 3 Department of Histology and Embryology, Wroclaw Medical University, Wroclaw, Poland; 4 Department of Physiotherapy, Wroclaw University School of Physical Education, Wroclaw, Poland; University of Insubria, Italy

## Abstract

It was recently proposed that UDP-galactose:ceramide galactosyltransferase (UGT8), enzyme responsible for synthesis of galactosylceramide (GalCer), is a significant index of tumor aggressiveness and a potential marker for the prognostic evaluation of lung metastases in breast cancer. To further reveal the role of UGT8 and GalCer in breast cancer progression, tumorigenicity and metastatic potential of control MDA-MB-231 cells (MDA/LUC) and MDA-MB-231 cells (MDA/LUC-shUGT8) with highly decreased expression of UGT8 and GalCer after stable expression of shRNA directed against UGT8 mRNA was studied in vivo in athymic nu/nu mice. Control MDA/LUC cells formed tumors and metastatic colonies much more efficiently in comparison to MDA/LUC-shUGT8 cells with suppressed synthesis of GalCer after their, respectively, orthotopic and intracardiac transplantation. These findings indicate that UGT8 and GalCer have a profound effect on tumorigenic and metastatic properties of breast cancer cells. In accordance with this finding, immunohistochemical staining of tumor specimens revealed that high expression of UGT8 accompanied by accumulation of GalCer in MDA-MB-231 cells is associated with a much higher proliferative index and a lower number of apoptotic cells in comparison to the MDA/LUC-shUGT8 cells. In addition, it was found that expression of UGT8 in MDA-MB-231 cells increased their resistance to apoptosis induced by doxorubicin in vitro. Therefore, these data suggest that accumulation of GalCer in tumor cells inhibits apoptosis, which would facilitates metastatic cells to survive in the hostile microenvironment of tumor in target organ.

## Introduction

In 1874 Thudichum isolated from bovine brain the lipid fraction that was highly enriched in galactosylceramide (then cerebroside) [1], for which the final structure was established in 1952 by Carter and Greenwood [2] and its enzymatic synthesis was described by Morrel and Radin in 1969 [3]. Since then, GalCer was primarily seen to be one of the major myelin stabilizing components [4]. This glycolipid, in addition to oligodendrocytes and Schwann cells, is also highly expressed in kidney and testis [5]–[6]. However, in contrast to many other glycosphingolipids, little is known about GalCer expression in human cancers except oligodendrogliomas and astrocytomas [7]–[8].

GalCer is synthesized by highly specific, reticulum-localized, glycosyltransferase UDP-ceramide:galactose galactosyltransferase (UGT8, EC 2.4.1.47) [9]. This enzyme is up-regulated in ER-negative breast cancer [10]–[11] and ovarian cancer as shown by microarray studies [12]. Using the same approach, UGT8 was listed as one of six genes predicting breast cancer lung metastases [13]. Recently, our studies with the use of immunohistochemistry and real-time PCR on the expression of UGT8 in breast cancer tissue specimens revealed significant increase in UGT8 expression in (1) metastatic vs. primary tumors, (2) tumors of malignancy grades G3 vs. G2 as well as G3 vs. G1 and (3) node-positive vs. node-negative tumors [14]. The predictive ability of increased expression of UGT8 was validated at the mRNA level in three independent cohorts of breast cancer patients. Therefore, our data suggested that UGT8 is a significant index of tumor aggressiveness and a potential marker for the prognostic evaluation of lung metastases in breast cancer. We also analyzed the presence of UGT8 and GalCer in breast cancer cell lines and found that cells with “luminal epithelial-like” phenotype did not express or weakly expressed UGT8 and GalCer, in contrast to malignant, “mesenchymal-like” cells forming metastases in nude mice [14].

GalCer is synthesized by transferring galactose to ceramide, which is the second messenger molecule involved in such basic cellular processes as induction of growth arrest, differentiation, senescence and apoptosis [15]. It is widely accepted that ceramide is part of specific signaling pathways related to cellular stress response and many stressors like cytokines, serum deprivation, heat shock, ionizing radiation, and chemotherapeutics generate enhanced ceramide production [16]. Among different ceramide activities, special attention was paid to the pro-apoptotic properties of this molecule [15], [17] as a potential target for cancer chemotherapy [18]. De novo synthesis is responsible for the accumulation of ceramide in receptor-dependent and receptor-independent induction of apoptosis in cancer cells by such chemotherapeutics as etoposides [19] or doxorubicin [20]. Using MCF-7 cells as a model, it was shown that ionizing radiation induces apoptosis of tumor cells by activating acid sphingomyelinase [21]. The same enzyme as well as neutral sphingomyelinase are involved in death receptor-mediated apoptosis of breast cancer cells [22]–[23]. On the other hand, ceramide, synthesized de novo or/and generated from other compounds, can be converted to several metabolites as ceramide 1-phosphate [24], sphingosine/sphingosine 1-phosphate [25], sphingomyelin [26], and 1-O-acylcermide [27]. Ceramide can also be glycosylated to form glucosylceramide (GlcCer) or GalCer. It is now well established that tumor cells, in order to escape apoptosis induced by various chemiotherapeutics and mediated by the accumulation of ceramide, convert this active lipid molecule to GlcCer [15], [28]–[29]. In contrast, little attention has been paid to an alternative ceramide glycosylation pathway by the formation of GalCer. Interestingly, few years ago, it was proposed, however without any experimental evidence, that accumulation of GalCer in tumor cells could inhibit apoptosis which facilitates metastatic cells to survive in the hostile microenvironment of the target organ [30]. It was also documented that the tumor microenvironment by itself is the source of numerous cell stresses, such as hypoxia, acidosis, hyperglycemia, hyperosmotic pressure, high cell density, and free radicals which affect cancer cells [31]. The involvement of UGT8 and GalCer in cellular stress response was shown in normal kidney cells subjected to hiperosmotic or hipertermic stresses. Such conditions generated UGT8 overexpression and synthesis of large amounts of GalCer as a way to decrease the level of pro-apotptotic ceramide [32]–[33]. Therefore, in the present work, to study the possible role of UGT8 and GalCer in the progression of breast cancer we created a specific cellular model, representing a “loss-of-function phenotype”, by transducing MDA-MB-231 with small hairpin (sh) RNA targeted UGT8 mRNA. The tumorigenic and metastatic properties of parental and sh-transduced MDA-MB-231 cells were compared in vivo, using athymic nude mice as the recipients of xenografted human breast cancer cell lines. The same cellular model was also used to study anti-apoptotic properties of GalCer in vitro.

## Materials and Methods

### Cells lines and culture conditions

Human breast cancer cell line MDA-MB-231 was purchased from the American Type Culture Collection (Manassas, VA, USA). All genetic manipulations described in this study were made within 1 month from the date of purchase. The cells were cultured in α-minimum essential medium (α-MEM) supplemented with 10% fetal calf serum (FCS; Cytogen), 2 mM L-glutamine, and antibiotics (complete α-MEM).

For apoptosis induction, breast cancer cells were grown in the presence of 0.005–0.5 µM or 2.5 µM doxorubicin (Ebewe) for 48 h, and for inhibition of ceramide glucosylation cells were treated with 5 µM DL-threo-1-phenyl-2-palmitoylamino-3-morpholino-1-propanol (PPMP, Sigma) for 96 h.

### Construction of silencing vector and lentivirus production

pLVTHM/LUC-shUGT8 silencing vector was obtained essentially as described by Solatycka et al. [34]. Briefly, shRNA/UGT8 targeted 1234–1252 bp of UGT8 mRNA (NM_001128174.1 and its 5′UTR variant: NM_003360.3). The DNA cassette consisted of H1 promoter and shDNA/UGT8 sequence was cloned into pLVTHM/LUC vector. To obtain pLVTHM/LUC vector, GFP cDNA was replaced by firefly luciferase cDNA (amplified from pGL3 plasmid by PCR) in pLVTHM lentiviral vector kindly provided as part of the lentivirus system by Dr. D. Trono (Ecole Polytechnique Fédérale de Lausanne, Switzerland).

For lentivirus production packaging LentiX 293T cells (Clontech) were co-transfected at 60% confluence with 40 µg of pLVTHM/LUC-shUGT8 or pLVTHM/LUC vector, 30 µg of psPAX2 and 10 µg of pMD2.G vectors (kindly provided by Dr. D. Trono, Ecole Polytechnique Fédérale de Lausanne, Switzerland) using polyethylenimine (Sigma-Aldrich) at a concentration of 0.05 mg/ml. After overnight incubation, the complete αMEM was replaced with fresh medium containing 1% FCS (Cytogen) and culture was continued for an additional 48 h. The virus-containing supernatant was filtered on a 0.22 µm syringe filter and concentrated 100× on Amicon Ultra-15K:100 000 (Milipore).

### Transductions

The MDA-MB-231 cells (2×10^4^) were infected with the concentrated virus stock (see above) by centrifugation (2460×g) at 23°C for 2 h. After overnight incubation, the medium was replaced with fresh complete α-MEM. The infection procedure was repeated twice every 5 days.

### Real-Time PCR assay

Total RNA was isolated from 10^6^ cells using RNeasy plus mini Kit (Qiagen) according to the manufacturer's recommendations. Reverse transcriptase reaction was performed using Super Script III First Strand synthesis Systems (Invitrogen). The relative amounts of mRNA were determined by quantitative real-time PCR with a iQ SYBR Green Supermix (BioRad) according to the manufacturer's instructions. The reaction mixtures for PCR contained, in addition to cDNA template, 10 pM of primers 5′-ugt8 (5′-CATGGTGTGCCTGTAGTGG-3′) and 3′-ugt8 (5′-GAGCCCTCTGACGGTAGC-3′) or 5′-gcs (5′-TGGATCAAGCAGGAGGACTTATAG-3′) and 3′-gcs (5′- TGTAGCAGGAAGCATGTTAATTCG-3′). For quantification, the samples were normalized against the expression of β-actin-encoding mRNA using the ΔΔC_T_ method. The specificity of the PCR was determined by melt-curve analysis for each reaction.

### SDS-PAGE and Western blotting

Cell lysates were obtained by treating the cell pellets with RIPA buffer (50 mM Tris/HCl pH 8.0, 150 mM NaCl, 1 mM EDTA, 0.5% NP-40) containing 1 mM phenylmethylsulphonyl fluoride (PMSF). The proteins were quantified by the bicinchonic acid protein assay kit (Sigma-Aldrich) and subjected to SDS/PAGE on 10–12% gel according to Laemmli. Separated proteins were transferred to nitrocellulose (BioRad) by wet electro-transfer. The blots were incubated with rabbit polyclonal antibodies directed against UGT8 (Sigma-Aldrich) or monoclonal antibodies (mAb) against caspase-3 (Cell Signaling) or β-actin (Abcam) overnight at 4°C. Anti- caspase-3 antibody detects full length caspase-3 (35 kDa) and the large fragment of caspase-3 resulting from cleavage (17 kDa). After washing with TBS containing 0.2% Tween 20 (TBST), the blots were incubated for 1 h at room temperature (RT) with horseradish-conjugated goat polyclonal antibodies directed against rabbit immunoglobulins (Dako) or horseradish-conjugated goat polyclonal antibodies against mouse immunoglobulins (Dako), respectively. After washing with TBST, the membrane was treated with a mixture consisting of Lumi-Light^PLUS^ Luminal/Enchancer Solution and Lumi-Light^PLUS^ Stable Peroxide Solution (Roche), and the BioLight film was developed by GBX Developer and an appropriate fixer (Kodak).

### Purification of neutral glycolipids and thin-layer chromatogram binding assay

Neutral glycolipids were purified as described previously [35] and analyzed by thin layer chromatography on silica gel 60 HP-TLC plates (Merck) with a solvent system of 2-propanol/15 M ammonia solution/methyl acetate/water, 75/10/5/15 by vol. [36]. Glycosphingolipids were detected with primuline solution (0.05% primuline in acetone/water 4∶1 by vol). GalCer was detected by TLC binding assay with rabbit polyclonal antibodies directed against GalCer (Sigma-Aldrich) and horseradish peroxidase-conjugated goat anti-rabbit IgG (Dako). The bound antibodies were visualized with Lumi-Light^PLUS^ (Roche) luminescence substrate. After luminescence light exposition the BioLight film was developed by GBX Developer and an appropriate fixer (Kodak).

### Metabolic labeling of cellular glycosphingolipids

Cells were grown for 3 h in the presence of 2 µCi/ml ^14^C-serine (Perkin-Elmer) in α-MEM supplemented with 1% FCS (Invitrogen). Cells were detached by 0.05% trypsin/0.02% EDTA, washed with PBS and pelleted. After purification and thin-layer chromatography, the neutral glycosphingolipids were detected by autoradiography. Samples of glycosphingolipids from metabolically labeled cells were exposed to radiographic screen (DuPoint) for 5 days.

### SRB and MTT assays

The cells (5×10^3^) were grown in 96-well plates (Saerstedt, Germany) in complete α-MEM. For SRB assay, after every 24 h, they were fixed by cold 10% trichloroacetic acid for 30 min in 4°C, washed with water and dried. The next day fixed cells were incubated with 0.4% sulforhodamine B (SRB, Sigma) in 1% acetic acid for 20 min in RT. After washing with 1% acetic acid, the protein-bound dye was extracted with 10 mM Tris. The absorbance at 492 nm was measured in EnSpire 2300 Multilabel Reader (Perkin-Elmer). Data were presented as means ±SD from two independent assays. For MTT assay, the next day, cells were incubated with increasing amounts of doxorubicin for 48 h. After this time, thiazolyl blue tetrazolium bromide (MTT) dissolved in PBS at a concentration of 5 mg/ml was added to each well, and the cells were cultured for an additional 2 h. The medium was removed and generated MTT-formazan was dissolved in DMSO. The absorbance at 570 nm was measured in a EnSpire 2300 Multilabel Reader (Perkin-Elmer). The experiments were performed in triplicate and repeated twice.

### Tumor growth and metastasis assays

Athymic Crl:NU-Foxn1^nu^ female mice, 8–10 weeks old, were obtained from Charles River (USA) and kept under pathogen-free conditions. Animal experiments were performed according to the International Animal Care Convention and all experimental procedures described in this study were approved by the First Local Ethic Committee for Animal Experimentation (Wroclaw, Poland). Human breast cancer cells were harvested with the use of 0.05% trypsin/0.02% EDTA, washed with PBS, and re-suspended in the same buffer. Cell suspensions (2×10^6^/100 µl PBS) were mixed with the same volume of ice-cold BD Matrigel Matrix High *Concentration* (Becton Dickinson) and the whole mixture was inoculated subcutaneously (s.c.). Tumor cell transplantations were performed under general anesthesia with isoflurane (Baxter). Tumor growth rate was monitored once a week by caliper measurements (starting from week two after cell inoculation), and tumor volume was calculated using the formula: (a^2^×b)/2, where a is the shorter diameter in mm and b is the longer diameter in mm. Body condition score (BCS) technique was used to assess health status of experimental mice as a part of daily routine. Eight weeks after breast cancer cells implantation the mice were sacrificed *by cervical dislocation after light anesthesia by isoflurane inhalation*. Within entire duration of the experiment, tumor diameters have not exceeded 15 mm. The tumor tissues were collected in 10% buffered formalin and were subjected to histological studies.

For metastasis assay, mice were inoculated intracardially (i.c.) under general anesthesia with isoflurane (Baxter). Breast cancer cells (2.5×10^5^) suspended in 100 µl of PBS were injected into the left ventricle using G26 needles under USG control (“Mylab 25 Gold”, Esaote). Developed metastases were monitored once a week by whole-body in vivo bioluminescence imaging using Night-Owl Nc100 imaging system (Berthold). Luciferase (2.25 mg/mouse) was injected into the peritoneum cavity 5 minutes before bioluminescence imaging. The same exposition time (5 min) was used for every image. For quantification of luminescence signals, regions of interest (ROI) (luminescence areas) were estimated automatically by WinLight32 software (Berthold) using the same threshold for every image. The number of photons per second from particular ROIs was calculated. Event-free survival was defined as the bioluminescence-free period. Body condition score (BCS) technique was used to assess health status of experimental mice as a part of daily routine. Mice with *cachexia* symptoms (BCS = 1) *were sacrificed by cervical dislocation after light anesthesia by isoflurane inhalation*. Internal organs were fixed in 10% buffered formalin for histological studies, processed routinely, and the slides were stained with hemotoxylin and eosin.

### Terminal Transferase dUTP Nick End Labeling (TUNEL) Assay

Apoptotic assay was performed using the ApopTag® Peroxidase In Situ Apoptosis Detection Kit (Millipore). Paraffin sections were de-waxed in xylene, rehydrated in alcohol, rinsed in distillate water and washed with PBS (pH 7.4). Then the sections were incubated with Proteinase K (Dako) for 5 min at RT and rinsed in PBS. Endogenous peroxidase was blocked by incubation in 3% H_2_O_2_/1xPBS for 5 min. Next, the sections were incubated, first with Equilibration Buffer for 10 min at RT and then with TdT Enzyme and Reaction Buffer at 37°C for 1 h. The reaction was stopped by the Stop Buffer and anti-dioksygenin peroxidase conjugated antibodies were applied for 30 min at RT. To visualize the TUNEL positive cell nuclei, the sections were incubated for 10 min with diaminobenzidine (Dako). Finally, the sections were counterstained with Mayer's hematoxylin and after dehydration in alcohols mounted in SUB-X Mounting Medium (both Dako). TUNEL-positive cell nuclei expression in tumour cells was evaluated under BX-41 light microscope, which had a computer assisted image analysis program Cell^D^ (both Olympus). Three fields with the highest number of tumour cells yielding positive reaction (hot spots) were selected for every stained section and analyzed. The general result for every section was the average of the three hot spot percentages of cells showing brown reaction product.

### Immunohistochemistry (IHC)

Tissue samples were fixed in 10% buffered formalin, dehydrated, and embedded in paraffin. For immunohistochemical staining, 4-μm-thick paraffin sections were cut. The sections were boiled in Target Retrieval Solution (pH 6 for Ki-67) using Pre-Treatment Link Platform (both Dako) and cooled in TBS/0.1% Tween. Then they were washed in Tris-buffered saline and incubated with monoclonal antibody against Ki-67 (MIB-1, Dako) for 30 min at RT in Link48 Autostainer (Dako). Bound antibodies were visualized using EnVision™ Detection Systems Peroxidase/DAB, Rabbit/Mouse (Dako). All slides were counterstained with Mayer's hematoxylin. The intensity of Ki-67 antigen was evaluated as described for TUNEL assay.

### Statistical analysis

All statistical analyses were performed using GraphPad Prism 5 software. The results were considered statistically significant when p≤0.05.

## Results

### GalCer inhibits tumor growth

**Figure 1:**
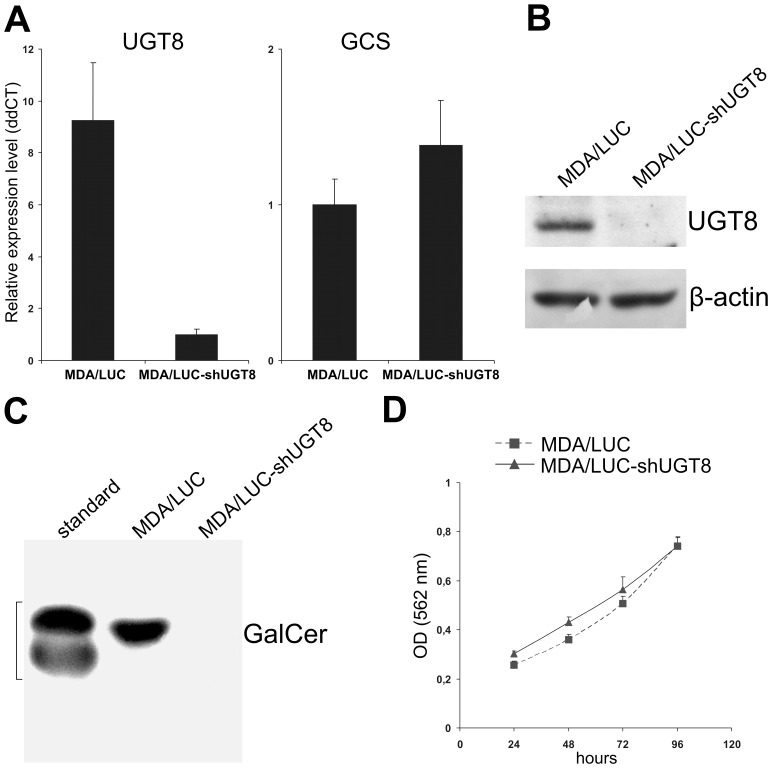
Characteristic of MDA-MB-231/LUC-shUGT8 cells with silenced UGT8 gene expression (A). Expression of UGT8 and GCS mRNAs in control MDA-MB-231 cells transduced with vector alone (MDA/LUC) and MDA-MB-231 cells tranduced with pLVTHM/LUC-shUGT8 construct (MDA/LUC-shUGT8). UGT8 and GCS levels were normalized against β-actin. (B) Western blot analysis of anti-UGT8 rabbit polyclonal antibodies binding to cellular proteins of control MDA/LUC and sh-transduced MDA/LUC-shUGT8 cells. Cell lysates, equivalent to 40 µg protein, were separated by SDS-PAGE under reducing conditions on a 10% gel and electrophoretically transferred onto a nitrocellulose membrane. β-Actin served as an internal control. (C) Immunostaining of neutral glycolipids from control MDA/LUC and sh-transduced MDA/LUC-shUGT8 breast cancer cell lines, separated by HP-TLC, with anti-GalCer rabbit polyclonal antibodies. For the analyzed cell lines, an aliquot of total neutral glycolipids corresponding to 1×10^7^ cells were applied to the HP-TLC plate. (D) proliferation of control MDA/LUC and sh-transduced MDA/LUC-shUGT8. Cell proliferation was determined using SRB assay as described in the “Materials and Methods”. The values are shown as the mean ± SD of eight independent replicates.

It was recently proposed that accumulation of GalCer in breast cancer cells inhibits apoptosis, which facilitates metastatic cells to survive in the hostile microenvironment of the target organ [14], [30]. To address this hypothesis we generated a specific loss-of-function phenotype by transducing MDA-MB-231 cells expressing UGT8 and GalCer with pLVTHM/LUC-shUGT8 construct in order to inhibit the expression of the enzyme, and in turn the synthesis of GalCer. Such cells, named MDA/LUC-shUGT8, were characterized by highly decreased levels of UGT8 mRNA (Fig. 1A) and highly decreased binding of anti-UGT8 antibodies to cell lysates in comparison to control MDA/LUC cells obtained after transduction of MDA-MB-231 cells with pLVTHM/LUC vector (Fig. 1B). Importantly, immunostaining of neutral glycosphingolipids purified from MDA/LUC-shUGT8 cells revealed highly decreased binding of anti-GalCer antibodies to TLC plates compared to MDA/LUC cells (Fig. 1C). It should be mentioned that the level of glucosylceramide synthase (GCS) mRNA was essentially the same in both cell types (Fig. 1A) as well as the morphology and growth rate (Fig. 1D).

**Figure 2:**
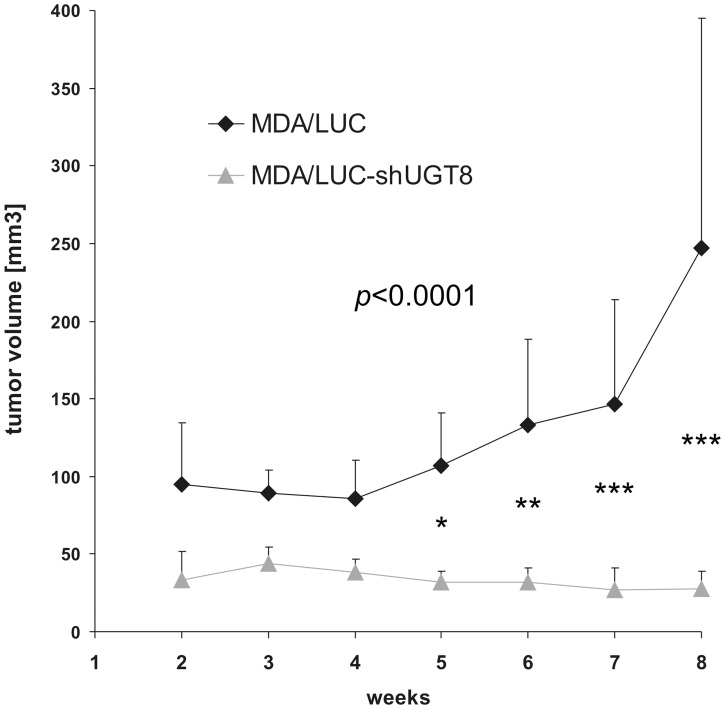
Xenograft tumor growth of MDA/LUC-shUGT8 cells with silenced expression of UGT8 gene and control MDA/LUC cells. Tumor growth was recorded on a weekly basis using metric calipers. Data are shown as the mean tumor volume for each group of mice (n = 7) ±SE at each indicated time point. (^***^p<0.0001, two-way ANOVA with Bonferroni posttests).

**Figure 3:**
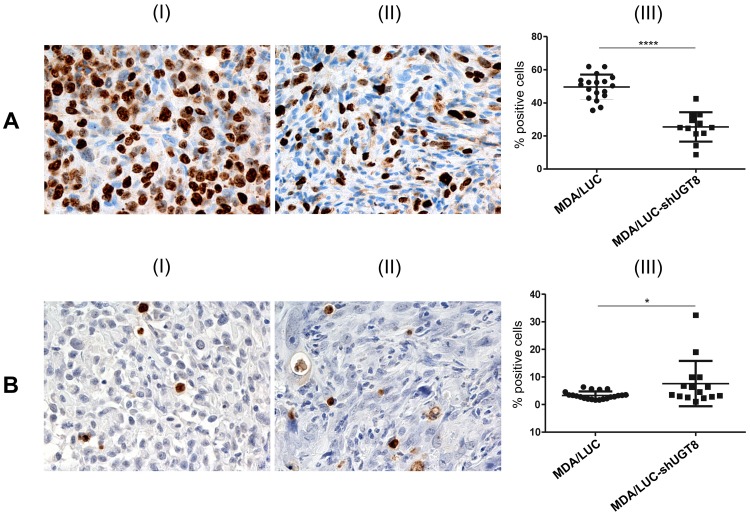
Immunohistochemical analysis of tumor xenografts of MDA/LUC-shUGT8 cells with silenced expression of UGT8 gene and control MDA/LUC cells. (A) Immunohistochemical staining with monoclonal antibody against Ki67 antigen of tumor sections after subcutaneous implantation of control MDA/LUC cells (I) and sh-transduced MDA/LUC-shUGT8 cells with decreased expression of UGT8 (II) into nu/nu mice. The numbers of Ki67-positive cells in MDA/LUC (I) and sh-transduced MDA/LUC-shUGT8 tumors were compared by Mann-Whitney U-test (^***^p<0.0001). (B) TUNEL technique after subcutaneous implantation of MDA-M/LUC cells (I) and MDA/LUC-shUGT8 cells (II) breast cancer cells into nu/nu mice. Original magnification: x100. The numbers of apoptotic cells in MDA/LUC (I) and sh-transduced MDA/LUC-shUGT8 tumors were compared by Mann-Whitney U-test (^*^p = 0.0432).

To assess the possible relationship between UGT8 expression and the malignant phenotype of breast cancer cells, MDA/LUC-shUGT8 and MDA/LUC cells were transplanted subcutaneously into nude mice. Five weeks after cancer cell transplantation, statistically important decreases in volumes were observed in the case of MDA/LUC-shUGT8 tumors in comparison to tumors formed by MDA/LUC cells (Fig. 2). It was found that at the end of the experiment (week 8), the mean volume of tumors formed by control MDA-MB-231 cells was 247.3 mm^3^ (±146.6). The mean tumor volume in mice injected with MDA/LUC-shUGT8 cells was 11% (27.4 mm^3^, ±11.5) of the total control tumor volume (p<0.0001, two-way ANOVA test) To determine if the observed differences in tumor volumes were associated with in vivo proliferative potential and apoptotic properties of breast cancer cells, MDA-MB-231/LUC and MDA-MB-231/LUC-shUGT8 tumors were subjected, respectively, to staining with mAb directed against Ki67 antigen and TUNEL assay. The percentage of Ki67-positive cells was reduced from 50% for MDA-MB-231/LUC cells to 25% in the case of MDA/LUC-shUGT8 cells (p<0.0001, Mann-Whitney U-test) (Fig. 3A). In contrast, when cancer cells were analyzed by TUNEL assay, an increased number of apoptotic cells was found in MDA/LUC-shUGT8 tumors as compared to MDA/LUC tumors (p = 0.0432, Mann-Whitney U-test) (Fig. 3B).

### GalCer affects metastasis formation

**Figure 4:**
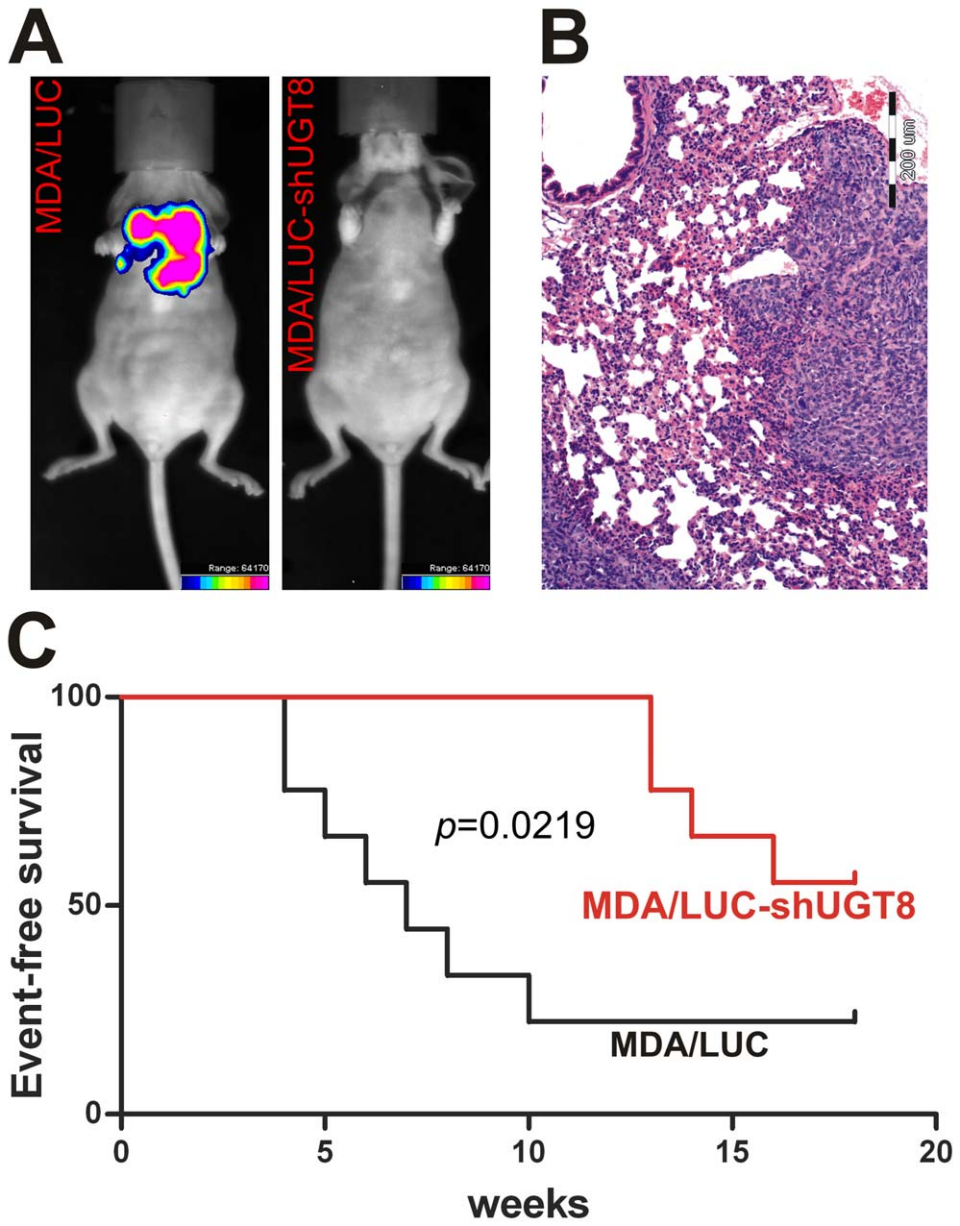
Metastatic potential of MDA/LUC-shUGT8 cells with silenced expression of UGT8 gene and control MDA/LUC cells. (A) Presence of metastases is shown by bioluminescence imaging in whole mice in week 9 of experiment. Breast cancer control MDA/LUC and MDA/LUC-shUGT8 cells (2.5×10^5^) with decreased expression of UGT8 were transplanted intracardially into athymic Crl:NU-Foxn1^nu^ mice and bioluminescence signal was measured in whole animal once a week. The intensity of bioluminescence emission is represents as a pseudocolor image. (B) Microscopic metastasis in lung after intracardiac implantation of MDA/LUC cells. H&Ex100 (C) Appearance of metastases after intracardiac implantation of MDA-MB-231/LUC and MDA-MB-231/LUC-shUGT8 defined as event-free survival. Event-free survival was defined as bioluminescence-free period after inoculation of breast cancer cells. n = 9 mice for each group. (^*^p = 0.0219, Mentel-Cox test).

In addition to tumorigenic potential, breast cancer cells with different expression of UGT8 and GalCer were studied for their metastatic potentials. After intracardiac inoculation of control MDA/LUC cells and MDA/LUC-shUGT8 cells with suppressed expression of UGT8 and GalCer, the formation of experimental metastases was monitored weekly by bioluminescence imaging (BLI) (Fig. 4A, Fig. S1) In the case of control MDA-MB-231 cells by the end of week 18, the presence of metastases was found in 73% of mice. Injection of MDA/LUC-shUGT8 cells resulted in reduced potential to form metastases in comparison to control cells. Tumors were found in 44% of mice. Results from BLI were further confirmed by histopathological analysis. Experimental metastases were found primarily in lungs (Fig. 4B), and in a few cases they were localized in brain and bones. It should be emphasized that in mice inoculated with MDA/LUC cells bioluminescence signals were detected from week 4, whereas in animals transplanted with MDA/LUC-shUGT8 cells such signals were not observed until week 13. The differences in metastatic potentials of MDA/LUC and MDA/LUC-shUGT8 cells were further assessed by comparison of bioluminescence signal-free-survival curves (Fig. 4C). Using Kaplan-Meier analysis it was found that mice with high expression of UGT8 had a shorter metastasis-free survival, referred as bioluminescence signal-free-survival, than animals with suppressed expression of UGT8 (p = 0.0219, Mentel-Cox test). In summary, our data indicates that suppression of UGT8 expression resulting in the absence of GalCer in breast cancer MDA-MB-231 cells has a profound effect on their tumorigenic properties and metastatic potential.

### UGT8 and GalCer expression correlates with resistance of breast cancer cells to doxorubicin-induced apoptosis

**Figure 5:**
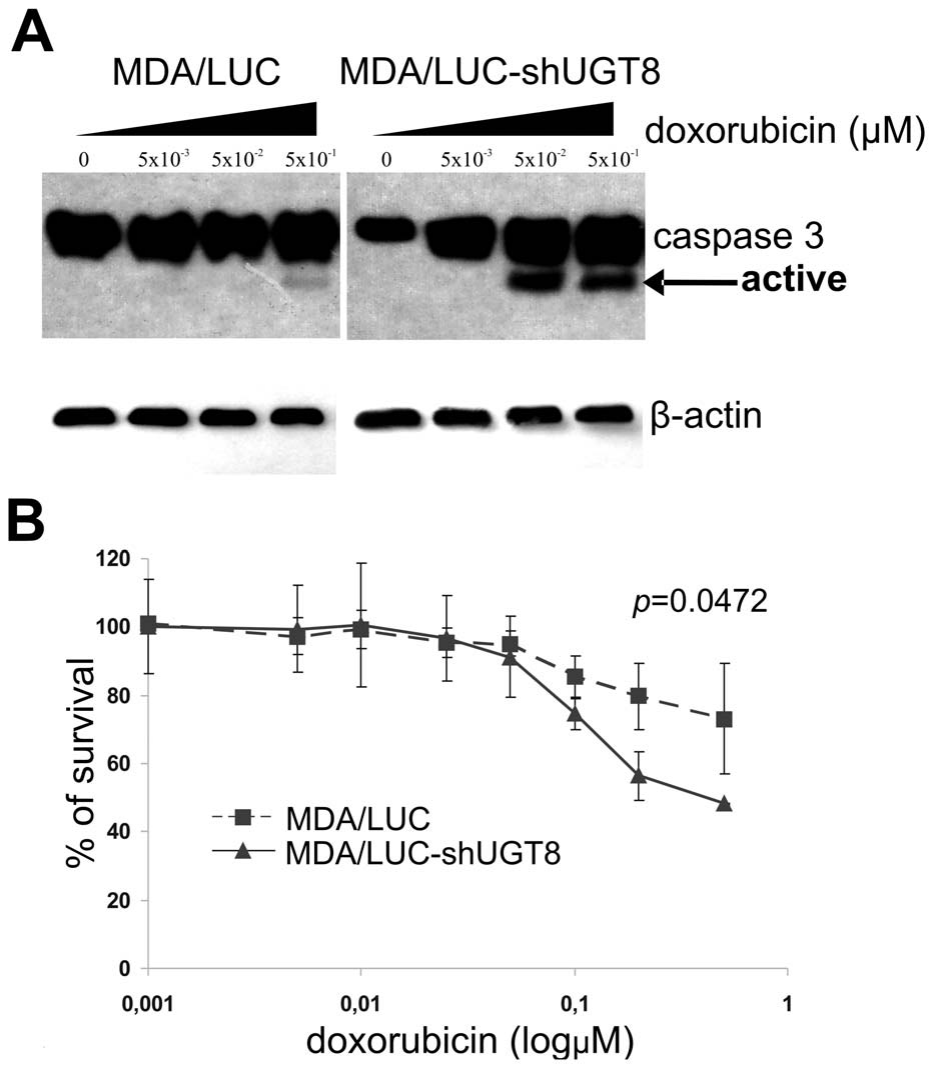
Response to doxorubicin of MDA/LUC-shUGT8 cells with silenced expression of UGT8 gene and control MDA/LUC cells. (A) Western blot analysis of anti-caspase-3 mAbs binding to cellular proteins of control MDA/LUC and MDA/LUC-shUGT8 cells with decreased expression of UGT8, grown at 70% confluence in the presence of increasing amounts of doxorubicin (0.005–0.5 µM) for 48 h. Cell lysates, equivalent to 40 µg protein, were separated by SDS-PAGE under reducing conditions on a 13% gel and electrophoretically transferred onto a nitrocellulose membrane. β-Actin served as an internal control. (B) Survival of control MDALUC cells and MDA/LUC-shUGT8 cultured in the presence of increasing concentrations of doxorubicin (0.001–1.0 µM) for 48 h. Cell viability was determined using MTT assay as described in the “Materials and Methods”. Data represent the mean ± SD of six replicates from two independent experiments. (^*^p = 0.0472, two-way ANOVA test).

It was previously shown that GalCer increases resistance of tumor cells to stress induced apoptosis [37] and its expression is elevated in multidrug resistant cells [38]–[39]. Therefore, to determine if GalCer affects stress induced apoptosis in breast cancer MDA-MB-231 cells, we chose doxorubicin as a pro-apoptotic agent which generates a high level of intracellular ceramide in tumor cells [40]–[42]. The MDA/LUC-shUGT8 cells with highly decreased levels of UGT8 and GalCer and control MDA/LUC were incubated with 0.005–0.5 µM doxorubicin for 24 h. After incubation with the drug, cells were analyzed by immunoblotting using anti-caspase-3 mAbs as an apoptosis indicator. This antibody recognizes inactive native protein (35 kDa) and fragments of caspase-3 resulting from its cleavage (17 kDa). It was found that both types of cells are resistant to apoptosis at a doxorubicin concentration of 0.005 µM, since only native caspase-3 was detected (Fig. 5A). At a concentration of 0.05 µM a small fragment of caspase-3 resulting from cleavage of native enzyme was seen only in MDA/LUC-shUGT8 cells. With higher concentrations of doxorubicin the presence of 17 kDa fragment of caspase-3 was also found in control MDA/LUC cells, but the intensity of the band was much weaker in comparison to MDA/LUC-shUGT8 cells. Therefore, our data revealed that breast cancer cells with low expression of UGT8 and GalCer are more sensitive to doxorubicin-induced apoptosis than control cells with high levels of UGT8 and GalCer expression. This was confirmed further by MTT assay. As shown in Fig. 5B, the viability of MDA/LUC cells was significantly higher than MDA/LUC-shUGT8 cells when exposed to increasing concentrations of doxorubicin (p = 0.0472, two-way ANOVA test) for 48 h.

**Figure 6:**
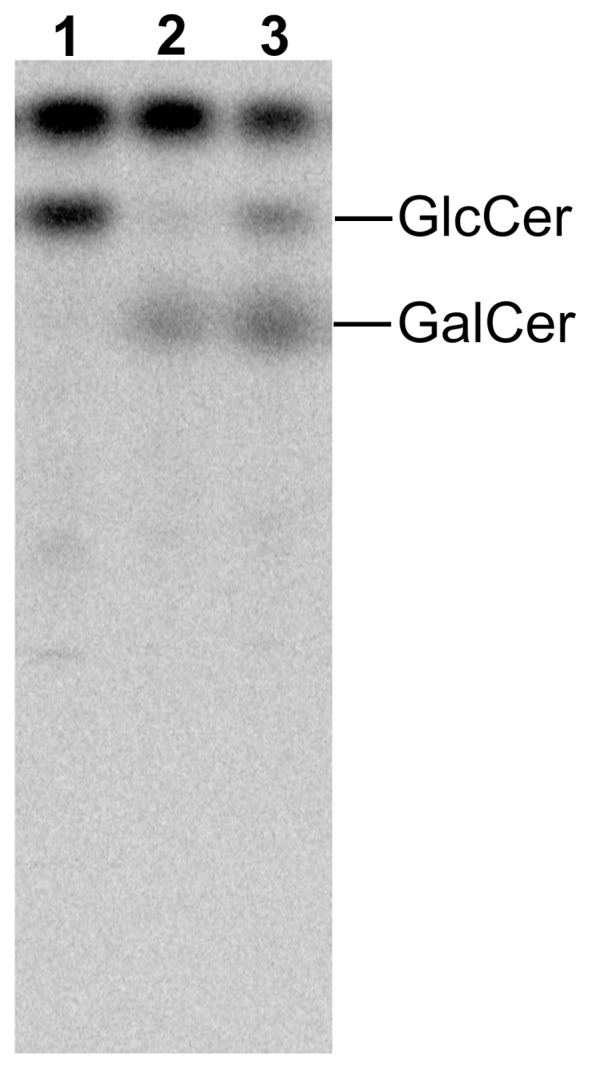
HPTLC pattern of ^14^C-serine-labelled neutral glycosphingolipids of the MDA-MB-231 cells after doxorubicin treatment. Cells were grown in medium containing 2 µC/ml ^14^C-serine for 3 h. Neutral glycosphingolipids were separated in the solvent system 2-propanol/15 M ammonia/methyl acetate/water 75/10/5/15 by vol. The plate was exposed to radiographic screen (DuPoint) for 5 days. The positions of GlcCer and GalCer standards are indicated on the right. Lane 1 – MDA-MB-231 cells grown in complete α-MEM, lane 2 - MDA-MB-231 cells grown in the presence of PPMP for 96 h, lane 3 – MDA-MB-231 cells grown at 90–100% confluence in the presence of 2.5 µM doxorubicin for 48 h.

To assess if the expression of GalCer in breast cancer cells can be directly affected by the presence of doxorubicin, the synthesis of this glycosphingolipid was analyzed in parental MDA-MB-231 metabolically labeled for 3 h with ^14^C-serine after 48 h incubation with 2.5 µM doxorubicin. The presence of doxorubicin resulted in the accumulation of GalCer (Fig. 6, lane 3) in contrast to control cells grown in the absence of the drug (Fig. 6, lines 1). Interestingly, an increase in GalCer synthesis was associated with a decreased level of GlcCer. To confirm that the observed changes are related specifically to GalCer synthesis, and not to changes in the synthesis of GlcCer, the MDA-MB-231 cells were incubated with 5 µM PPMP, a potent GlcCer synthesis inhibitor, for 96 h. The presence of this compound inhibited only the appearance of band migrating as GlcCer standard (Fig. 6, lane 2).

## Discussion

During malignant transformation cancer cells undergo profound changes in sphingolipid metabolism resulting in abnormal expression of such metabolites as ceramide, sphinganine, sphingosine, sphingosine-1-phosphate, GalCer and enzymes: acid ceramidase, sphingosine kinase 1, S1P lyase, S1P phosphatase, ceramide kinase, serine palmitoyl-coA transferase, alkaline sphingomyelinase, GCS, and UGT8 [29]. However, information about GalCer and UGT8 in cancer cells is scarce. Based on microarray data it was shown that elevated expression of UGT8 gene in breast cancer was significantly associated with ER-negativity and more malignant tumors [11]. UGT8 is one of six genes whose elevated level correlated with a significantly increased risk of lung metastases in breast cancer patients [13]. This latter finding was confirmed on the level of UGT8 protein [14]. In the same study, it was proposed that UGT8 is a significant index of tumor aggressiveness and a potential marker for the prognostic evaluation of lung metastases in breast cancer. Interestingly, it was also found that metastatic breast cancer cell lines (MDA-MB-231, MCF10CA1a.cl1) are characterized by highly elevated levels of UGT8 and GalCer in comparison to non-metastatic ones (MCF7, T47D, SKB-3, BT-474).

Based on the results obtained for clinical samples and breast cancer cell lines, the present study was undertaken to evaluate the role of UGT8 and GalCer in tumorigenic and metastatic properties of breast cancer cells. It was done by constructing specific “loss-of-function” phenotype represented by MDA-MB-231 cells transduced with small hairpin (sh) RNA targeted UGT8 mRNA (MDA/LUC-shUGT8 cells). The role of UGT8 and GalCer in tumor growth and formation of experimental metastases by control MDA-MB-231 cells and MDA/LUC-shUGT8 cells was studied in vivo in athymic nu/nu mice. Control MDA/LUC cells formed tumors much more efficiently in comparison to MDA/LUC-shUGT8 cells with suppressed synthesis of GalCer after their orthotropic transplantation. It should be stressed, that in experiments related to another project, MDA-MB-231 cells transduced with the same vector carrying small hairpin RNA targeting different protein and transplanted into nu/nu mice formed tumors of the same mean volume as control MDA/LUC cells, suggesting that off-target effects of shRNA targeting UGT8 mRNA are unlikely (results not shown). In accordance with the above finding, immunohistochemical staining of tumor specimens revealed that high expression of UGT8 accompanied by accumulation of GalCer in MDA-MB-231 cells is associated with a much higher proliferative index and a lower number of apoptotic cells in comparison to the MDA/LUC-shUGT8 cell line. It was also found that breast cancer cells expressing higher levels of UGT8 and synthesizing larger amounts of GalCer revealed a higher ability to form metastatic colonies after intracardiac inoculation into nu/nu mice. Both of these facts indicate that the suppression of UGT8 expression in MDA-MB-231 cells has a profound effect on their tumorigenic and metastatic properties. Our data agree with the recent results of Li et al., who found that expression of UGT8 was closely associated with metastatic potential of human pancreatic cancer cells in nude mice model [43]. However, there is no data showing how changes in the expression of UGT8 can possibly affect formation of metastases by these cells. According to Beier and Gorogh (2005) accumulation of GalCer in tumor cells inhibits apoptosis, which facilitates metastatic cells to survive in the hostile microenvironment of tumors in target organs [30]. It is well documented that the tumor microenvironment is the source of numerous cell stresses, such as hypoxia, acidosis, hyperglycemia, hyperosmotic pressure, high cell density, and free radicals which affect cancer cells [31]. The cells, including cancer cells, respond to environmental forces by developing cellular stress response and/or cellular homeostasis response [44]. Therefore, cellular stress is considered an important factor in tumorigenesis and metastasis formation [45]. One of the widely recognized stress indicators in living cells, involved in such important cellular processes as induction of growth arrest, differentiation, senescence and apoptosis, is ceramide [15]–[16]. This simple molecule acts as a cellular rheostat controlling cell fate, either inducing apoptosis or promoting cell survival [46]. The intracellular ceramide level can be regulated by de novo synthesis, sphingomyelin and glycosphingolipids catabolism/synthesis or by dephosphorylation of such metabolites as ceramide-1-phosphate [29]. It was shown that glycosylation of ceramide by GCS, resulting in the synthesis of GlcCer and reduction of ceramide in drug-resistant leukemia and cancer cells, protects them from apoptosis induced by doxorubicin [47]–[50]. Based on these results, it is now widely accepted that accumulation of GlcCer in cancer cells caused by overexpression of GCS attenuates the accumulation of ceramide and contributes to drug resistance in multidrug-resistant cancer cells [29]. However, it was also shown that inhibition of GCS using D,L-threo-1-phenyl-2-decanoylamino-3-morpholino-1-propanol (PDMP) or 1-phenyl-2-palmitoylamino-3-morpholino-1-propanol (PPMP) in drug-sensitive U937 and HL60 cells actually protect them from daunorubicin-induced apoptosis [37]. Interestingly, such treatment did not increase intracellular ceramide concentrations but instead increased GalCer levels. Furthermore, in cells enriched in exogenous GalCer daunorubicin-induced apoptosis was significantly inhibited. Similarly, Krabbe cells with high levels of GalCer were more resistant to daunorubicin- and cytosine arabinoside-induced apoptosis in comparison to Gaucher cells with lower levels of GalCer. Therefore, to elucidate the anti-apoptotic properties of GalCer in drug-sensitive breast cancer cells, MDA/LUC cells and MDA/LUC-shUGT8 cells were incubated with doxorubicin, and the cells were analyzed by Western blotting for the presence of caspase-3. It was found that under stress conditions induced by doxorubicin, control MDA-MB-231 cells (MDA/LUC) are more resistant to apoptosis in comparison to MDA-MB-231 cells (MDA/LUC-shUGT8) with suppressed expression of UGT8, which do not synthesize GalCer. This agrees with the results obtained for Krabbe cells with high levels of GalCer and Gaucher cells with lower levels of GalCer [37]. The involvement of GalCer, and not the GlcCer, in the resistance of MDA-MB-231 cells to doxorubicin-induced apoptosis is supported by the fact that in parental MDA-MB-231 cells grown in the presence of doxorubicin and metabolically labeled with ^14^C-serine a pronounced increase of GalCer level was associated with a concomitant decrease in GlcCer synthesis. In addition, it was shown that in relatively doxorubicin-resistant MDA-MB-231 cells [51], the expression of GCS mRNA is approximately 10-fold lower than in doxorubicin-sensitive MCF7 cells [50], which are devoid of GalCer [14]. Also, doxorubicin treatment does not affect the expression of GCS in MDA-MB-231 in contrast to up-regulation of GCS in MCF7 cells [50], which is also true for drug-sensitive HL-60 cells and drug-resistant HL-60/ADR cells [47]. Interestingly, the increased expression of UGT8 resulting in accumulation of GalCer was observed in Madin-Darby canine kidney cells in response to hyperosmotic and heat stresses [32]–[33].

The question remains as to the exact mechanism/mechanisms by which GalCer mediates cytoprotective effects during breast cancer progression and stress-induced apoptosis in drug-sensitive breast cancer cells. At this point of our research we can only speculate how this glycosphingolipid may affect the biological functions of these cells, taking into account previous studies on GalCer and GlcCer. It was shown that increased conversion of ceramide to GlcCer, lowering the intracellular pool of ceramide may confer the ability of MDR cells to escape from avoid apoptosis [52]. Similarly, increased expression of UGT8 and GCS under heat and hyperosmotic stresses in MDCK cells increased both GalCer and GlcCer and decreased ceramide content helping them to escape from cell apoptosis [32]–[33]. On the other hand, blocking GCS in drug-sensitive leukemic U937 cells did not lead to an increase in daunorubicin-induced ceramide production, but instead increased ceramide galactosylation [37]. According to the authors, cell sensitivity to drug-induced apoptosis depends on the intracellular balance between GalCer and GlcCer. We did not determine ceramide levels directly. However, our results suggest that regardless of ceramide levels, increase of galactosylceramide is a pro-survival mechanism for breast cancer cells. Research is in progress to define exact mechanisms by which GalCer protects breast cancer cells from apoptosis.

## Supporting Information

Figure S1 Presence of metastases in athymic Crl:NU-Foxn1nu mice transplanted with breast cancer control MDA/LUC and MDA/LUC-shUGT8 cells with silenced expression of UGT8 gene in 3^rd^, 6^th^ and 9^th^ week of experiment. Metastases were detected by bioluminescence imaging. Breast cancer cells (2.5×10^5^) were transplanted intracardially and biolumiencsence signal was measured in whole animal once a week. The intensity of bioluminescence emission is represented as a pseudocolor image.
(TIF)
