# Supplementary figures and images for: Galactosylceramide Affects Tumorigenic and Metastatic Properties of Breast Cancer Cells as an Anti-Apoptotic Molecule
Source: PLoS One. 2013 Dec 31;8(12):e84191. doi: 10.1371/journal.pone.0084191 (PMC3877204; doi:10.1371/journal.pone.0084191)

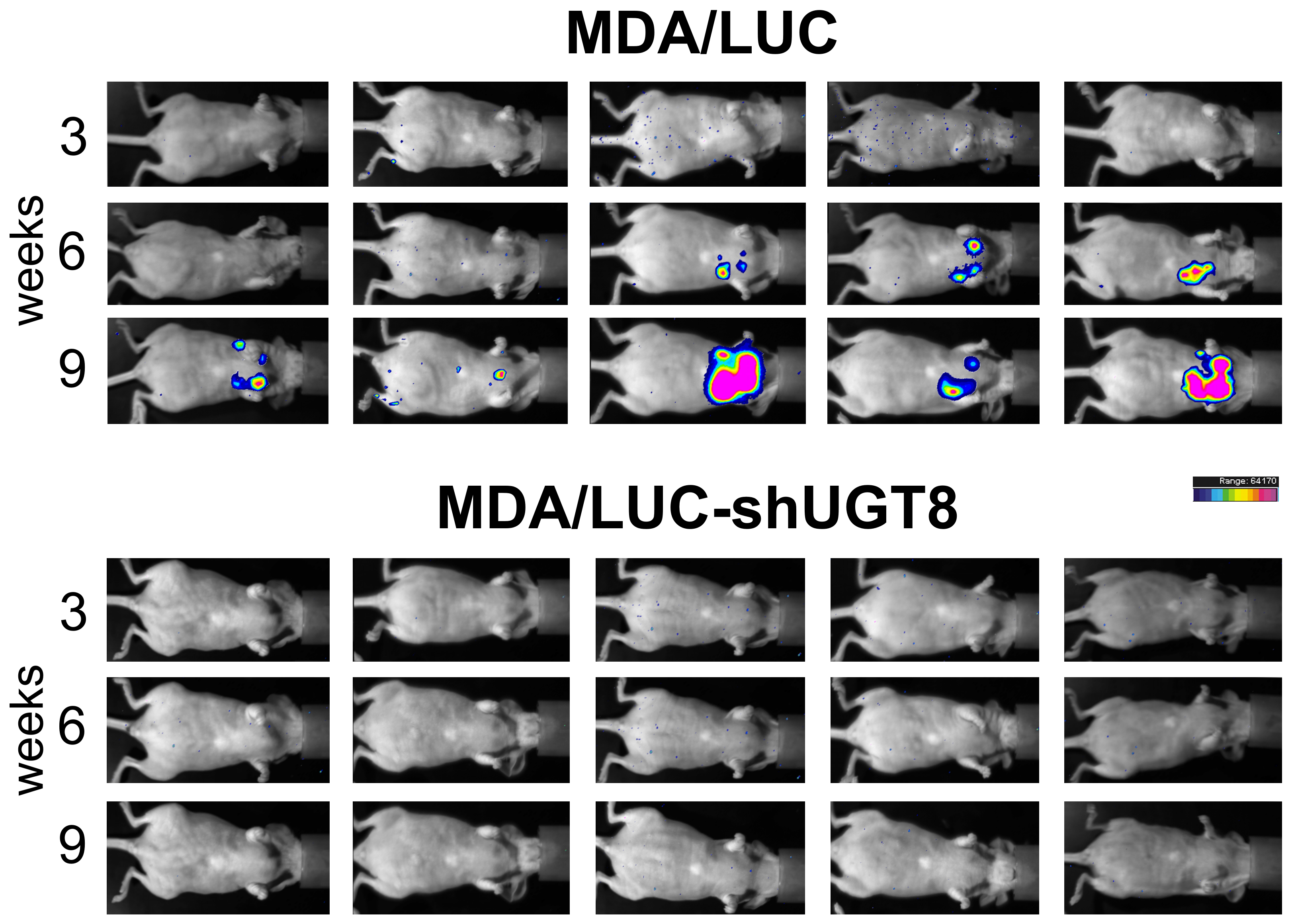

Supplement: Figure S1 — Presence of metastases in athymic Crl:NU-Foxn1nu mice transplanted with breast cancer control MDA/LUC and MDA/LUC-shUGT8 cells with silenced expression of UGT8 gene in 3rd, 6th and 9th week of experiment. Metastases were detected by bioluminescence imaging. Breast cancer cells (2.5×10^5^) were transplanted intracardially and biolumiencsence signal was measured in whole animal once a week. The intensity of bioluminescence emission is represented as a pseudocolor image. (TIF) [file pone.0084191.s001.tif]
